# Comparative in silico characterization of *Klebsiella pneumoniae* hypervirulent plasmids and their antimicrobial resistance genes

**DOI:** 10.1186/s12941-022-00514-6

**Published:** 2022-06-02

**Authors:** Negin Bolourchi, Anam Naz, Maryam Sohrabi, Farzad Badmasti

**Affiliations:** 1 Department of Bacteriology, Pasteur Institute of Iran, Tehran, Iran; 2 Institute of Molecular Biology and Biotechnology (IMBB), The University of Lahore (UOL), Lahore, Pakistan; 3 Microbiology Research Center (MRC), Pasteur Institute of Iran, Tehran, Iran

**Keywords:** *Klebsiella pneumoniae*, Hypervirulent plasmid, Antimicrobial resistance gene

## Abstract

**Background:**

The hypervirulent pathotype of *Klebsiella pneumoniae* (hvKp) is mainly mediated by large virulent plasmids. It seems that these hypervirulent plasmids (HVPs) are accumulating antimicrobial resistance genes (ARGs) and are turning quickly into drug-resistant hypervirulent hybrids. Therefore, molecular mechanisms involved in this convergence needs to be investigated to control their global spread.

**Methods:**

In this study, the complete sequence of 79 non-redundant hypervirulent plasmids were retrieved from GenBank and their genetic features, hypervirulence and antimicrobial resistance patterns (AMR) as well as their putative transmission capability were compared using bioinformatics tools.

**Results:**

The majority of HVPs belonged to clonal complex (CC)23, and sequence type (ST)11. IncFIB and IncHI1B were the most prevalent plasmid replicon types. Out of 79 plasmids, 78 were positive for *iutA* and *iucA*. The *iucC*, *iucB* and *iucD* genes were found in 77 plasmids. Almost 26% of the HVPs were potentially conjugative of which 71% carried AGRs. ARGs against beta-lactams, carbapenems, quinolones, aminoglycosides, chloramphenicols, tetracyclines and macrolides were detected in 30% of HVPs. Class 1 integron and prophage structures harboring multiple ARGs were found in eight plasmids. Insertion sequences (IS)*6*, IS*110* and IS*1380* appeared to be important genetic elements in transmission of ARGs.

**Conclusions:**

The high prevalence of *iucA* and *iutA* suggests their strong capability for rapid and accurate genetic markers for discrimination of hvKp in the laboratory. This study indicated the important role of mobile genetic elements (MGEs) in the emergence of drug-resistance in hypervirulent strains. The high prevalence of putative conjugative hybrids implies higher incidence of multidrug-resistant (MDR)-hvKp strains in near future.

**Supplementary Information:**

The online version contains supplementary material available at 10.1186/s12941-022-00514-6.

## Introduction

Unlike classical *Klebsiella pneumoniae* as a major nosocomial opportunistic with high level of antimicrobial resistance (AMR); hypervirulent *K. pneumoniae* (hvKp) is a highly virulent pathogen and sensitive to antimicrobials [1]. It has the ability to cause community-acquired infections including liver abscesses, meningitis, endophthalmitis, necrotizing fasciitis, osteomyelitis as well as pneumonia [2]. The clinical outcome of hvKp are severe and mortality rate reaches 40% in blood-stream infections [3].

The hypervirulent pathotype of *K. pneumoniae* is attributed to the carriage of virulence genes involved in iron acquisition, hyper capsule production and heavy metal resistance [4, 5]. This pathotype is mainly mediated by large hypervirulent plasmids (HVPs) [6, 7]. Despite multiple investigations on molecular mechanisms involved in hypervirulence of *K. pneumoniae*, a comprehensive study on extra-chromosomal hypervirulence reservoirs has not been conducted yet.

The importance of HVPs increases when they encode antimicrobial resistance genes (ARGs) and turn into drug-resistant hypervirulent hybrids [8]. Previously, the prevalence of drug-resistant hvKp strains was low, however; reported cases have been increased worldwide and become a new threat [9]. Several studies from Europe, South America, Australia, and North America have reported MDR-hvKp [10]. Even though various ARGs have been detected in clinical hvKp strains, our knowledge on the role of HVPs in transmission of AMR genetic determinants among hypervirulent strains is still limited. The continuous accumulation of ARGs particularly in wide-spread sequence types (ST) of hvKp is troublesome. This is even more life-threatening in case of carpabememase-producers [11]. The mortality rate of carbapenem-resistant hvKp sepsis has been reported as high as 100% [12]. In 2021, an extensively drug-resistant hvKp positive for *bla*_NDM_ and *bla*_OXA−48_ was reported from a neonatal hospital [13]. Therefore, it is highly urgent to characterize AMR genetic reservoirs of HVPs to predict the future behavior of hvKp regarding antibiotic therapy.

In this analytical study, we characterized and compared the hypervirulence pattern of 79 pre-assembled HVPs of different *K. pneumonia* STs as well as their ARGs using bioinformatic tools. In addition, the plasmids were investigated for their replicon types, conjugation capability, plasticity and phylogenetic relationship.

## Materials and methods

### Preparation of initial dataset

The term “hypervirulent *K. pneumoniae”* was searched in the GenBank database (https://www.ncbi.nlm.nih.gov/genbank). The complete nucleotide sequence of all hypervirulent chromosomes and their plasmids were retrieved from GenBank.

### Clonal relatedness of strains harboring HVPs

The distribution of HVPs among different hvKp STs were investigated. For each plasmid, the ST of its related chromosome based on seven housekeeping genes (*gapA*, *infB*, *mdh*, *pgi*, *phoE*, *rpoB* and *tonB*) was determined using the PubMLST database (https://pubmlst.org/) [14]. To characterize the clonal relationship of hvKp strains, the minimum spanning tree of all hvKp STs was prepared using PHYLOViZ version 2.0 [15].

### Circular alignment and phylogenetic analysis of HVPs

To compare plasmids sequence plasticity, circular alignment was performed for several HVPs of major STs using the BLAST Ring Image Generator (BRIG) version 0.95 [16]. To investigate the phylogenetic relationship of the plasmids, a Neighbor Joining (NJ) tree was constructed based on genes with occurrence of > 10% in all plasmids using BacCompare (http://baccompare.imst.nsysu.edu.tw) [17]. The cladogram was visualized using iTOL (https://itol.embl.de) [18].

### Genetic characterization of HVPs

The plasmids size was determined using DFAST (https://dfast.ddbj.nig.ac.jp/) [19]. The putative conjugation capability of plasmids was determined by using the MOB-suite software [20]. The presence of conjugal apparatus including *oriT*, relaxase, type IV coupling protein (T4CP) and type IV secretion system (T4SS) was investigated using oriTfinder (https://bioinfo-mml.sjtu.edu.cn/oriTfinder/) [21]. The incompatibility (Inc) group of plasmids was determined using MOB-suite.

### Characterization of hypervirulence determinants

The encoding sequence of 14 genes involved in hypervirulent phenotype including *iroB, iroC, iroD, iroN*, *iucA, iucB, iucC, iucD, iutA* (aerobactin siderophores), *peg-589* and *peg-344* (metabolite transport), *terB* (tellurite resistance), *rmpA* and *rmpA2* (hypermucoviscosity) [22] were retrieved from the NCBI database (https://www.ncbi.nlm.nih.gov/genbank) and blasted against all HVPs to determine their hypervirulence profile using the BLASTn software [23].

### Antimicrobial resistance analysis of HVPs

All ARGs on plasmids were identified using the comprehensive antibiotic resistance database (CARD) (https://card.mcmaster.ca/analyze/rgi) [24]. To investigate the role of mobile genetic elements (MGEs) in distribution of ARGs among hvKp strains, the plasmids were submitted to PHASTER (http://phaster.ca/) to detect prophage structures [25]. The identified prophages then were submitted to ISfinder to determine the presence of insertion sequences (ISs) and transposon elements (https://isfinder.biotoul.fr/) [26]. The presence of integrons was investigated using BLASTn. The ARGs cassette arrays surrounding the integron genes were characterized using DFAST and ORFfinder (https://www.ncbi.nlm.nih.gov/orffinder/).

## Results

### Clonal relatedness of strains harboring HVPs

**Fig. 1:**
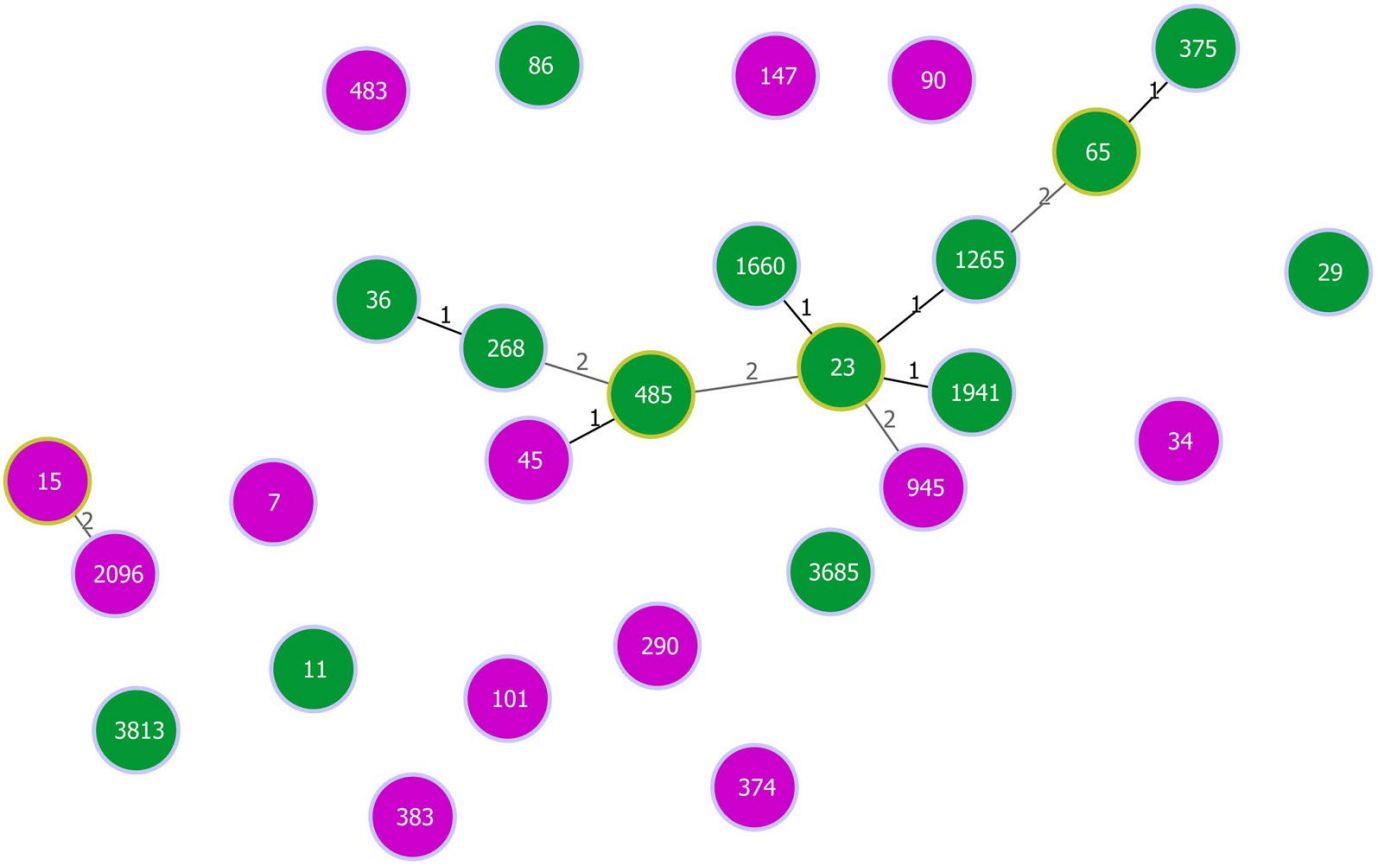
The minimum spanning tree of STs carrying the *Klebsiella pneumoniae* hypervirulent plasmids with similarity cut off of > 4 allelic types (green: STs carrying all 14 hypervirulent genes, purple: STs not carrying all hypervirulent genes). Clonal complex 23 (CC23) including ST23, ST1941, ST1660, ST485, ST268, ST36, ST1265, ST65, and ST375 harbored complete hypervirulence pattern. While, non-CC23 including ST11, ST3813, ST86, and ST29 lack several hypervirulence genes

Seventy-nine non-redundant HVPs were found in GenBank. Their corresponding hvKp strains were belonged to clonal complex (CC)23 (including ST23, ST485, ST1660, ST1265, ST1946, ST268, ST36, ST65 and ST375) as well as single clonal lineages ST7, ST11, ST15, ST29, ST34, ST147, ST86, ST101, ST90, ST374, ST383, ST3685, ST83 and ST3913. See Fig. 1. Six plasmids were found in strains with undetermined ST. See Additional file 1: Table S1.

### Genetic plasticity and phylogenic analysis and of HVPs

**Fig. 2:**
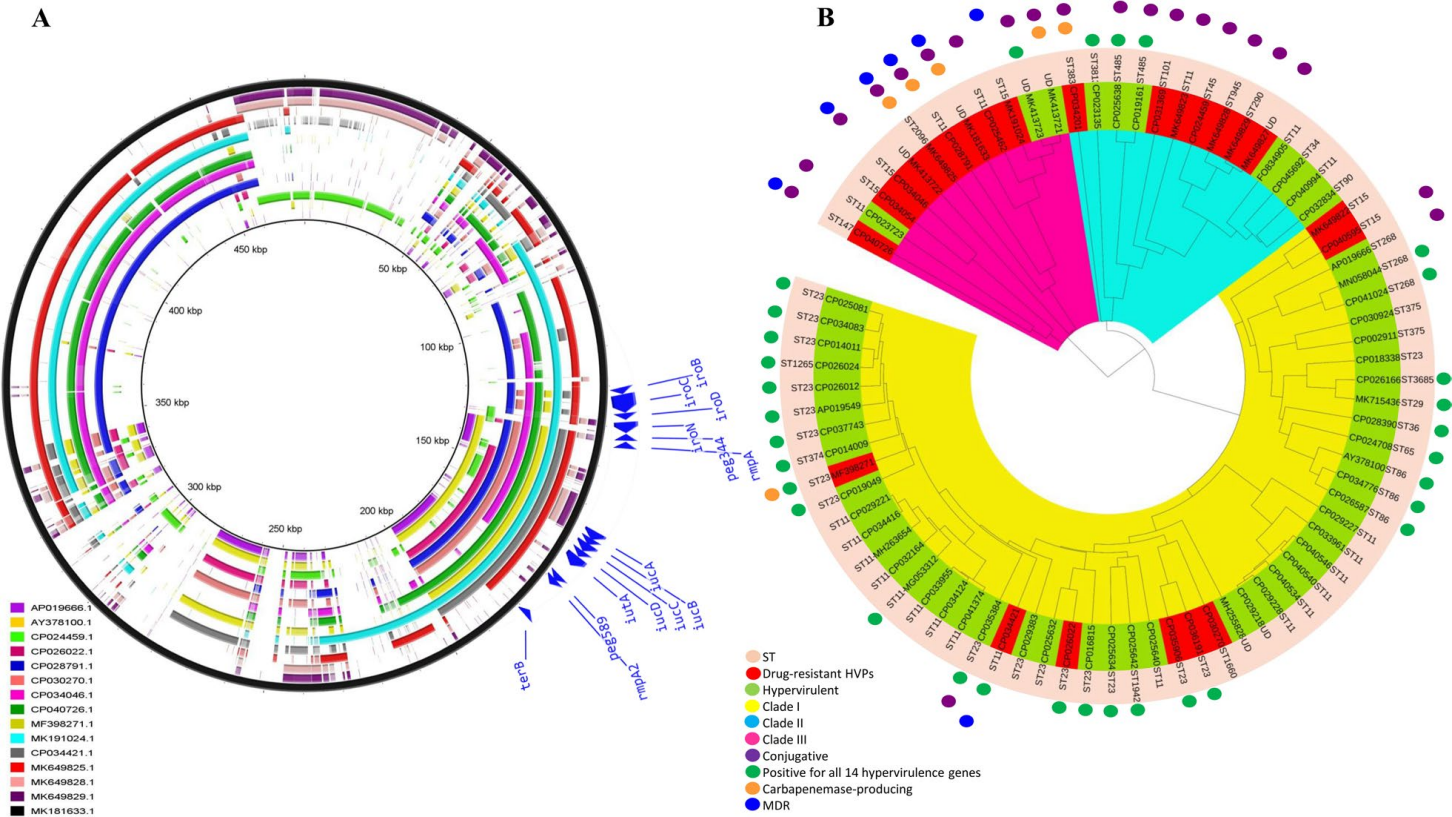
**A** The multiple circular alignment of 15 K*. pneumoniae* hypervirulent plasmids. High heterogenicity was detected among plasmids. However, they were similar in some regions. **B** The phylogenetic NJ dendrogram of 79 K*. pneumoniae* hypervirulent plasmids and distribution of drug-resistant hybrids. The subclade distribution of plasmids within the leaves was not exactly associated with their strains STs. Conjugative antimicrobial-resistant hybrids were mainly distributed in clade II and III. Besides, almost all MDR-HVPs were located in clade III. While, clade I mainly contained non-drug-resistant HVPs

High heterogeneity was observed among different HVPs. However, they have similarity in some regions regardless of their strains STs. See Fig. 2A.

A number of 2952 of genes with > 10% occurrence rate were identified for plasmids by BacCompare. The results showed that the HVPs had 1589 unique genes. The heatmap of 79 plasmids showed that the plasmids have different allele numbers in 185 loci. See Additional file 3: Fig. S1. The phylogenetic dendrogram based on 2952 gene classified the HVPs in three main clades (I, II and III). See Fig. 2B.

### Genetic characteristics of HVPs

The size of plasmids ranged from 96,085 to 479,335 bp. Twenty-one plasmids were potentially conjugative carrying all four conjugal constituents including *oriT*, relaxase, type IV coupling protein (T4CP) and type IV secretion system (T4SS). Three plasmids including CP034421.1, CP045692.1 and CP032834.1 were mobilizable (only lacked *oriT*). All plasmids were positive for T4CP and T4SS.

**Fig. 3:**
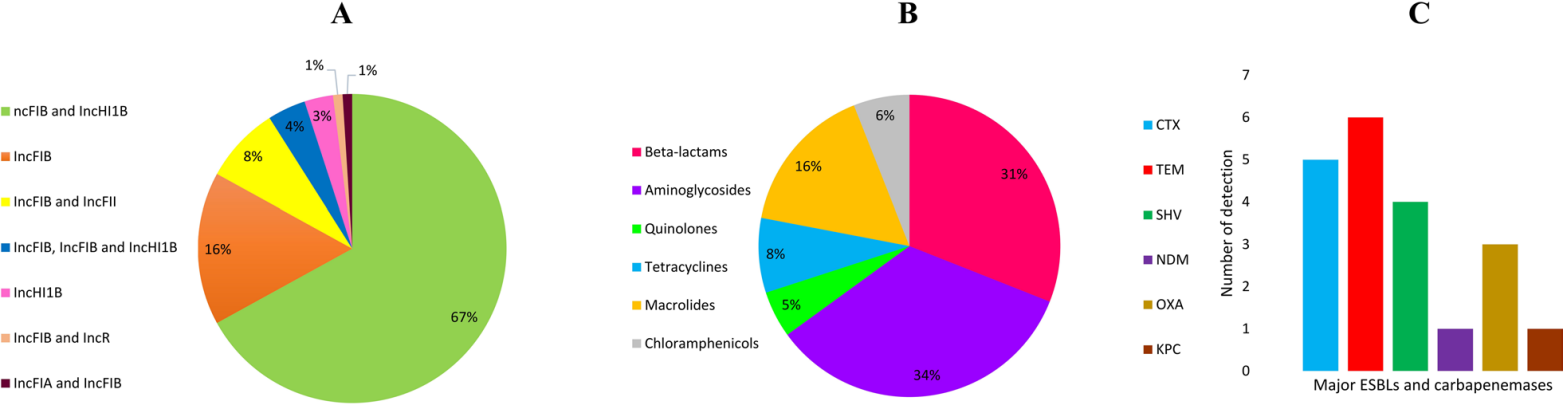
**A** The prevalence of different Inc groups among 79 K*. pneumoniae* hypervirulent plasmids. IncFIB was the most abundant (97%) replicon type among HVPs which co-existed with IncHI1B in 70% of the plasmids. **B** The prevalence of ARGs *K. pneumoniae* hypervirulent plasmids against different drug classes. The plasmids were mainly resistant to beta-lactamand aminoglycosides modifying enzymes. **C** The frequency of major ESBLs and carbapenemases. *bla*_TEM_, *bla*_CTX-M_ and *bla*_SHV_ were the most abundant ESBL genes among hypervirulent plasmids

A number of 56 plasmids had IncFIB and IncHI1B. Thirteen plasmids had only IncFIB. A number of seven plasmids had the IncFIB and IncFII replicon types. CP025640.1 and CP029227.1 had only IncHI1B. MK181633.1, CP035384.1 and MF398271.1 had three replicon types (IncFIB, IncFIB and IncHI1B). IncFIB and IncR were detected in CP045692.1. CP040994.1 had IncFIA and IncFIB. See Additional file 1: Table S1 and Fig. 3A.

### Hypervirulence profile

**Fig. 4:**
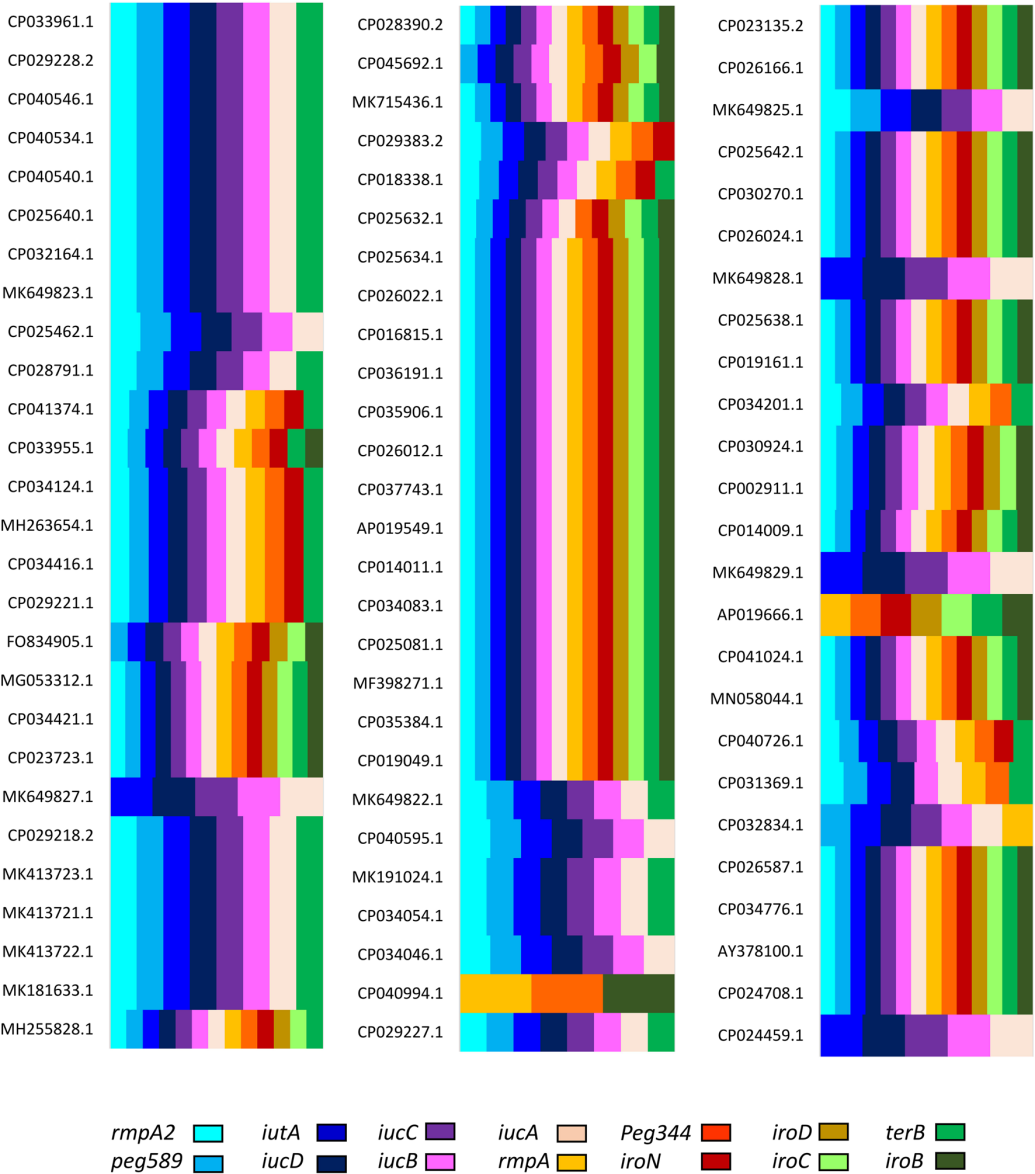
The hypervirulence profile (*iroB, iroC, iroD, iroN*, *iucA, iucB, iucC, iucD, iutA, terB*, *rmpA*, *rmpA2, peg-589* and *peg-*344) of 79 K*. pneumoniae* hypervirulent plasmids. The *iucA*, *iutA*, *iucD*, *iucB*, *iucC*, *rmpA2*, *peg-589* and *terB* genes were highly prevalent among plasmids

A number of 32 plasmids carried all 14 hypervirulence related genes including *iroB, iroC, iroD, iroN*, *iucA, iucB, iucC, iucD, iutA, terB*, *rmpA*, *rmpA2, peg-589* and *peg-344*. Out of 79 plasmids, 78 were positive for *iutA* and *iucA*. The *iucC*, *iucB* and *iucD* were found in 77 plasmids. The *rmpA2*, *peg-589* and *terB* genes were detected in 73, 70 and 66 HVPs, respectively. *peg-344* and *iroN* were identified in 51 and 49 plasmids, respectively. A number of 41 plasmids were positive for *iroD*, *iroC* and *iroB* genes*.* The fewest number of hypervirulence genes was for CP040994.1 (*peg-344*, *iroB* and *rmpA*). The schematic comparison of plasmids hypervirulence profile has been presented in Fig. 4.

### Antimicrobial resistance associated genetic elements in HVPs

#### ARGs

A number of 24 plasmids carried different ARGs against extended-spectrum beta-lactams (*bla*_TEM-1_, *bla*_DHA-1_, *bla*_CTX-M-15_, *bla*_CTX-M-24_, *bla*_SHV-11_, *bla*_SHV-64_, *bla*_SHV-5_ and *bla*_CMY-6_), carbapenems (*bla*_OXA-1_, *bla*_KPC-2_ and *bla*_NDM-1_), quinolones (*qnrB4*, *qnrB17* and *qnrS1*), aminoglycosides (*armA, aph(3'')-Ib*, *aph(3')-Ia*, *aac(6')-Ib-cr6*, *aac(6′)-lb-cr5*, *aac(6')-Ib10*, *aac(6′)-lb*, *aac(3)-lld*, *aac(3)-IIe*, *rmtC* and *ant(3'')-IIa*), chloramphenicol (*catI*, *catII*, *cmlA1*, and *catB3*), tetracycline (*tetA*, *tetB*, *tetD*, and *tetR*) and macrolide (*mphE*, *msrE*, and *mphA*). See Additional file 1: Table S1.

Quinolone resistance genes were identified in four plasmids including MK649823.1, MK191024.1, MK649829.1, and MK181633.1. Macrolide, tetracycline and chloramphenicol resistance genes were detected in 12, six, and five plasmids, respectively. A number of 25 and 23 aminoglycosidase and beta-lactamase encoding genes were found, respectively. See Fig. 3B. *bla*_CTX-M_, *bla*_TEM_ and *bla*_SHV_ were carried by six, five and four plasmids, respectively. CP028791.1 (*bla*_OXA-1_), MK181633.1 (*bla*_OXA-1_), MK649825.1 (*bla*_OXA-1_), MF398271.1 (*bla*_KPC-2_) and CP034201.1 (*bla*_NDM-1_) were carbapenemase producers. See Fig. 3C. Six plasmids were MDR-HVPs. See Additional file 1: Table S1.

#### Mobile genetic elements associated with AMR

##### Prophages

A number of 13 HVPs had prophage structures with Salmon_SJ46 and Escher_RCS47 being the most prevalent. The *bla*_CTX-M_ and *bla*_TEM-1_ genes were major ESBLs carried by prophage structures. *bla*_OXA-1_ of CP028791.1 was detected in Escher_RCS47. Genes encoding aminoglycosides modifying enzymes in MK181633.1, CP034046.1, CP034054.1, CP040595.1 and CP028791.1 carried by different incomplete prophages (including Bacill_Shanette, Salmon_SJ46, Escher_RCS47 and Escher_Av). Some ARGs against other antimicrobial drug classes including quinolones (*qnrS1)*, tetracycline (*tetA*) and chloramphenicol (*catB3*) were also phage-mediated. Prophages in five plasmids did not carry any ARGs. See Additional file 2: Table S2.

##### Integrons

Class 1 integron (*intI1*) was found in eight plasmids including CP034421.1, CP040595.1, CP040726.1, MK649825.1, CP030270.1, CP028791.1, CP034046.1, and MK181633.1. See Additional file 3: Fig. S2. ARGs which were found around class 1 integron were as follows: *aac(3)-lld*, *aac(6’)-lb* and *aac(6’)-lb-cr5* on plasmids CP034421.1, CP040595.1 and CP028791.1, respectively; *dfrA5* on CP040726.1, MK649825.1 and MK181633.1; *cmlA* and *catB3* on CP030270.1 and CP028791.1, respectively and *arr3* on CP028791.1. Class 2 integron (*sat2*) was detected only on CP034046.1

##### Insertion sequences and transposons

The IS*26* (IS*6*-like), IS*Vsa5* (IS*4*-like), IS*4321* (IS*110*-like), IS*Cfr1* (IS*1182*-like), IS*Ec9* (IS*1380*-like), IS*Kpn11*(IS*3*-like), IS*1326* (IS*2*-like), IS*110*, IS*1* elements as well as Tn*As3* (Tn*3*-like) and Tn*3* family transposases were truncated by *intI1* and multiple ARGs. See Additional file 3: Fig. S2. IS*6*, IS*110* and IS*1380* were frequently found on prophages surrounding beta-lactamase and other ARGs. See Additional file 3: Table S2.

## Discussion

Up to now, various genetic markers have been dedicated to hvKP [27]. However, the definition of hvKP is still not fully established due to the complexity of virulence mechanisms [28]. The present study was focused on plasmid-mediated mediators of hypervirulence. We considered genes involved in iron acquisition, metabolite transport, tellurite resistance and hypermucoviscosity as markers of hypervirulence. However, hypermucoviscose has been considered a pathotype distinct from hypervirulent in some studies [29, 30]. Moreover, the chromosomal carriage of genes such as *entB*, *mrkD*, *fimH*, *wabG*, *ybtS*, *allS* and *kfu* has been also investigated as hypervirulence mediators in several studies [3, 8, 30].

In consistent with other studies, CC23 was the most widely distributed CC among hvKp strains [31]. However, ST11 is the most prevalent clone in China [32]. In our study, 33% of hvKp strains belonged to ST11. Almost 41% of the HVPs were positive for all 14 hypervirulence genes of which 46% were isolated from CC23 and the remaining from ST11, ST286, ST375, ST86 and ST65. See Fig. 1. The hypervirulence patterns of strains belonging to a same ST were highly similar. In this regard, Yu et al*.* indicated that hvKp strains belonging to ST23, ST65, ST375 and ST86 were all positive for *rmpA*, *rmpA2*, *iutA*, and *iroN* on their plasmids.

Several studies have been conducted to find a method for accurate identification of hvKp in the laboratory [33]. The string test has been considered as a phenotypic marker for hvKp. However, its results are variable and sometimes unspecific [34]. Tellurite resistance was suggested as a screening method [33, 35]. Hypervirulent populations were successfully separated based on their capsules using density-gradient centrifugation test [36]. Wu et al. distinguished hvKp based on positive string test and positive *rmpA* and *iucA* [37]. Molecular detection of *rmpA* and *iucA* was also used as hypervirulent indicator by Li et al. [3]. The *peg-344*, *iroB*, *iucA*, *rmpA* and *rmpA2* genes showed > 95% diagnostic accuracy for identification of hvKp strains in 2018. The *peg-344, iucA* and *iutA* were introduced as the best markers for diagnosis of hvKp [38]. Based on the present virulence analysis, almost all HVPs harbored *iucA* and *iutA.* Therefore, molecular detection of *iucA* and *iutA* genes with accuracy of 98% can be advantageously used for precise screening of hvKp. The *iucD*, *iucB*, *iucC*, *rmpA2*, *peg-589* and *terB* genes were also highly prevalent among HVPs (with the prevalence rate of 97%, 97%, 97%, 92%, 88% and 83%, respectively). This highlights their important role in hypervirulent pathotype of *K. pneumoniae*.

HvKp and classical *K. pneumoniae* have two distinct phenotypes with similar clonal lineages [27]. Their convergence can be mediated by the genetic integration of drug-resistant plasmids in HVPs vice versa. Another probable mechanism is the transmission of ARGs in HVPs through recombinational processes. Also, it can result from the co-existence of antibiotic-resistant plasmids along with HVPs in one individual bacterium [39, 40].

IncFIB was the most abundant (97%) replicon type among HVPs which co-existed with IncHI1B in 70% of the plasmids. The presence of specific Inc groups in majority of plasmids suggests that the formation of convergent phenotype is rarely result from plasmids integration whereas is more related to other two mentioned mechanisms. Also, it suggests that a drug-resistant plasmid with Inc groups other than IncFIB and IncHI1B has higher chance to reside in a single hvKp bacterium.

The AGRs were detected in 30% of the HVPs. MK181633.1, MK413722.1, CP025462.1, CP034046.1, CP026022.1, CP040726.1, MK649829.1 and MK649825.1 were important due to the carriage of major ESBLs. Similar to classical plasmids, *bla*_TEM_ and *bla*_CTX-M_ were the most prevalent beta-lactamase producers among HVPs [41]. Almost 26% of the HVPs were potentially conjugative. See Fig. 2B. Interestingly, 71% of all putative conjugative HVPs carried AGRs. MK181633.1 was a conjugative MDR-HVP carrying the highest number of beta-lactamases and aminoglycosidases-producing genes. Of four carbapenem-resistant plasmids, three (MK181633.1, MK649825.1 and CP034201.1) harboring *bla*_OXA-1_ and *bla*_NDM-1_ were potentially conjugative. See Fig. 2B. Importantly, *bla*_KPC-2_ was carried by MF398271.1, a HVP harboring all 14 hypervirulent genes. Also, all quinolone resistance genes (*qnrB4*, *qnrB17* and *qnrS1*) were carried by potentially conjugative HVPs. The isolation of self-transmissible HVPs carrying major ARGs suggests the increase of resistance against most clinically used antimicrobials by a few years.

Similar to classical plasmids, class 1 integron is highly responsible for resistance to multiple drug classes in hvKp strains [42]. Importantly, our data showed that resistance to third-generation cephalosporins and aminoglycosides is mediated by prophages structures in some hypervirulent strains. The extra-chromosomal phage-mediated carriage of ESBLs in hvKp has been previously reported by Yang et al. [43]. Moreover, IS*6*, IS*110* and IS*1380* seem to be important elements for horizontal transmission of major ARGs [44].

A specific distribution of antimicrobial-resistant HVPs was observed in the NJ dendrogram. The subclade distribution of plasmids within the leaves was not exactly associated with their strains STs. Conjugative antimicrobial-resistant HVPs were mainly distributed in clade II and III. Besides, almost all MDR-HVPs were located in clade III. While, clade I mainly contains non-drug-resistant HVPs. See Fig. 2B. Importantly, the results exhibited lower presence of hypervirulent genes in clade II and III. Based on our data, majority of highly antimicrobial-resistant HVPs lacked *iroN*, *iroD*, *iroC*, *iroB* and *peg344*. By contrast, the full carriage of hypervirulent genes was mainly observed in plasmids belonging to clade I. This suggests that integration of antibiotic-resistant mediators in HVPs specifically occurs in plasmids with low levels of hypervirulence which are phylogenetically distinct from highly virulent plasmids to some extent.

## Conclusion

We conducted a comprehensive molecular analysis on plasmids-borne mechanisms of hypervirulence. Based on the present analysis, molecular detection of the *iucA* and *iutA* genes can be advantageously utilized for accurate identification of hvKp. Moreover, we characterized the AMR genetic reservoirs of HVPs. Several ARGs against beta-lactams, carbapenems, quinolones, aminoglycosides, chloramphenicols, tetracyclines and macrolides were detected in HVPs. This study highlighted the role of MGEs in the emergence of AMR among hvKp strains. The presence of potentially conjugative MDR-HVP hybrids in this study implies higher incidence of drug-resistant hvKp strains in near future.

## Supplementary Information

**Additional file 1: Table S1.** The genetic information, hypervirulence and antimicrobial resistance genetic patterns of 79 hypervirulent plasmids of *Klebsiella pneumoniae.*

**Additional file 2: Table S2**. The information of prophages found in hypervirulent plasmids of *Klebsiella pneumoniae*.

**Additional file 3. Fig. 1. **The ARGs cassettes surrounding class 1 integron in *Klebsiella* *pneumoniae* hypervirulent plasmids. **Fig. 2.** The phylogenic tree and heatmap of 79 hypervirulent *Klebsiella* *pneumoniae* plasmids based on 185 loci.

## Data Availability

The sequence of all 79 HVPs are available in the GenBank database.

## Abbreviations

AMR: Antimicrobial resistance
ARG: Antimicrobial resistant gene
CARD: Comprehensive antibiotic resistance database
ESBLs: Extended-spectrum beta-lactamase
HVPs: Hypervirulent plasmids
hvKp: Hypervirulent *Klebsiella pneumoniae*
MDR: Multidrug-resistant
MGE: Mobile genetic element
Inc: Incompatibility
IS: Insertion sequence
NJ: Neighbor joining
T4CP: Type IV coupling protein
T4SS: Type IV secretion system
ST: Sequence type
