# Supplementary material for: Comparative in silico characterization of Klebsiella pneumoniae hypervirulent plasmids and their antimicrobial resistance genes
Source: Ann Clin Microbiol Antimicrob. 2022 Jun 2;21:23. doi: 10.1186/s12941-022-00514-6 (PMC9161459; doi:10.1186/s12941-022-00514-6)
Supplement: Supplementary file 1 — Additional file 1: Table S1. The genetic information, hypervirulence and antimicrobial resistance genetic patterns of 79 hypervirulent plasmids of Klebsiella pneumoniae. [file 12941_2022_514_MOESM1_ESM.docx]

**Table S1**. The genetic information, hypervirulence and antimicrobial resistance genetic patterns of 79 hypervirulent plasmids of *Klebsiella pneumoniae.*

| No. | Plasmid name | Accession number | Size (bp) | *rmpA2* | *peg589* | *iutA* | *iucD* | *iucC* | *iucB* | *iucA* | *rmpA* | *peg344* | *iroN* | *iroD* | *iroC* | *iroB* | *terB* | Replicon type | Conjugation | *oriT* | Relaxase | T4SS | T4CP | MLST (ST) | Antimicrobial resistance genes |
| --- | --- | --- | --- | --- | --- | --- | --- | --- | --- | --- | --- | --- | --- | --- | --- | --- | --- | --- | --- | --- | --- | --- | --- | --- | --- |
| 1 | pSH9-VIR | MH255828.1 | 188437 | 1 | 1 | 1 | 1 | 1 | 1 | 1 | 1 | 1 | 1 | 1 | 1 | 0 | 1 | IncFIB and IncHI1B | Non-Mobilizable | 0 | 0 | 1 | 1 | UD | - |
| 2 | unnamed | MK181633.1 | 479335 | 1 | 1 | 1 | 1 | 1 | 1 | 1 | 0 | 0 | 0 | 0 | 0 | 0 | 1 | IncFIB IncFII, and IncHI1B | Conjugative | 1 | 1 | 1 | 1 | UD | *bla*_OXA-1_*_,_ bla*_CTX-M-15_*, bla*_TEM-1_*_,_ bla*_SHV-11_*_,_ mphE, mphA, msrE, armA, sul1, bla*_DHA-1_*, qnrB4, sul1, qacEdelta1, aadA2, dfrA12, aac(6')-Ib-cr6, tet(A), aph(3')-Ia* |
| 3 | p332306-HI3 | MK413722.1 | 317970 | 1 | 1 | 1 | 1 | 1 | 1 | 1 | 0 | 0 | 0 | 0 | 0 | 0 | 1 | IncFIB and IncHI1B | Non-Mobilizable | 0 | 0 | 1 | 1 | UD | *catI, bla*_SHV-64_*, tet(B), tetR* |
| 4 | p504051-HI3 | MK413721.1 | 270105 | 1 | 1 | 1 | 1 | 1 | 1 | 1 | 0 | 0 | 0 | 0 | 0 | 0 | 1 | IncFIB and IncHI1B | Conjugative | 1 | 1 | 1 | 1 | UD | - |
| 5 | p721005-HI3 | MK413723.1 | 269204 | 1 | 1 | 1 | 1 | 1 | 1 | 1 | 0 | 0 | 0 | 0 | 0 | 0 | 1 | IncFIB and IncHI1B | Conjugative | 1 | 1 | 1 | 1 | UD | - |
| 6 | p3-L201 | CP029218.2 | 177991 | 1 | 1 | 1 | 1 | 1 | 1 | 1 | 0 | 0 | 0 | 0 | 0 | 0 | 1 | IncFIB and IncHI1B | Non-Mobilizable | 0 | 0 | 1 | 1 | UD | - |
| 7 | p1675474_1 | MK649827.1 | 181647 | 0 | 0 | 1 | 1 | 1 | 1 | 1 | 0 | 0 | 0 | 0 | 0 | 0 | 0 | IncFIB and IncFII | Conjugative | 1 | 1 | 1 | 1 | 7 | *sul2, catII* |
| 8 | Unnamed | CP023723.1 | 297984 | 1 | 1 | 1 | 1 | 1 | 1 | 1 | 1 | 1 | 1 | 1 | 1 | 1 | 1 | IncFIB and IncHI1B | Conjugative | 1 | 1 | 1 | 1 | 11 | - |
| 9 | pVir-CR-hvKP-C1398 | CP034421.1 | 274540 | 1 | 1 | 1 | 1 | 1 | 1 | 1 | 1 | 1 | 1 | 1 | 1 | 1 | 1 | IncFIB and IncHI1B | Mobilizable | 0 | 1 | 1 | 1 | 11 | *aac(3)-IId, aadA5, qacEdelta1, sul1, mphA, dfrA17* |
| 10 | pVir-CR-HvKP267 | MG053312.1 | 233470 | 1 | 1 | 1 | 1 | 1 | 1 | 1 | 1 | 1 | 1 | 1 | 1 | 1 | 1 | IncFIB and IncHI1B | Non-Mobilizable | 0 | 0 | 1 | 1 | 11 | - |
| 11 | plasmid II | FO834905.1 | 121703 | 0 | 1 | 1 | 1 | 1 | 1 | 1 | 1 | 1 | 1 | 1 | 1 | 1 | 0 | IncFIB | Mobilizable | 0 | 1 | 1 | 1 | 11 | - |
| 12 | p1-L388 | CP029221.1 | 217870 | 1 | 1 | 1 | 1 | 1 | 1 | 1 | 1 | 1 | 1 | 0 | 0 | 0 | 1 | IncFIB and IncHI1B | Non-Mobilizable | 0 | 0 | 1 | 1 | 11 | - |
| 13 | pVir-CR-hvKP-C789 | CP034416.1 | 215950 | 1 | 1 | 1 | 1 | 1 | 1 | 1 | 1 | 1 | 1 | 0 | 0 | 0 | 1 | IncFIB and IncHI1B | Non-Mobilizable | 0 | 0 | 1 | 1 | 11 | - |
| 14 | pKPN-QL24 | MH263654.1 | 215940 | 1 | 1 | 1 | 1 | 1 | 1 | 1 | 1 | 1 | 1 | 0 | 0 | 0 | 1 | IncFIB and IncHI1B | Non-Mobilizable | 0 | 0 | 1 | 1 | 11 | - |
| 15 | p1b1 | CP034124.1 | 200216 | 1 | 1 | 1 | 1 | 1 | 1 | 1 | 1 | 1 | 1 | 0 | 0 | 0 | 1 | IncFIB and IncHI1B | Non-Mobilizable | 0 | 0 | 1 | 1 | 11 | - |
| 16 | p2_L39 | CP033955.1 | 198087 | 1 | 1 | 1 | 1 | 1 | 1 | 1 | 1 | 1 | 1 | 0 | 0 | 1 | 1 | IncFIB and IncHI1B | Non-Mobilizable | 0 | 0 | 1 | 1 | 11 | - |
| 17 | pKP58-1 | CP041374.1 | 197415 | 1 | 1 | 1 | 1 | 1 | 1 | 1 | 1 | 1 | 1 | 0 | 0 | 0 | 1 | IncFIB and IncHI1B | Non-Mobilizable | 0 | 0 | 1 | 1 | 11 | - |
| 18 | pOXA1_020030 | CP028791.1 | 288222 | 1 | 1 | 1 | 1 | 1 | 1 | 1 | 0 | 0 | 0 | 0 | 0 | 0 | 1 | IncFIB | Conjugative | 1 | 1 | 1 | 1 | 11 | *sul1, qacEdelta1, arr-3, catB3, bla*_OXA-1_*, aac(6')-Ib-cr6* |
| 19 | p44-1 | CP025462.1 | 261706 | 1 | 1 | 1 | 1 | 1 | 1 | 1 | 0 | 0 | 0 | 0 | 0 | 0 | 0 | IncFIB and IncHI1B | Conjugative | 1 | 1 | 1 | 1 | 11 | *mphA, bla*_TEM-1_*, aac(3)-IId* |
| 20 | pBA6740_1 | MK649823.1 | 226590 | 1 | 1 | 1 | 1 | 1 | 1 | 1 | 0 | 0 | 0 | 0 | 0 | 0 | 1 | IncFIB and IncFII | Conjugative | 1 | 1 | 1 | 1 | 11 | *qnrB17* |
| 21 | unnamed1 | CP032164.1 | 207409 | 1 | 1 | 1 | 1 | 1 | 1 | 1 | 0 | 0 | 0 | 0 | 0 | 0 | 1 | IncFIB and IncHI1B | Non-Mobilizable | 0 | 0 | 1 | 1 | 11 | - |
| 22 | pKP9 | CP025640.1 | 190470 | 1 | 1 | 1 | 1 | 1 | 1 | 1 | 0 | 0 | 0 | 0 | 0 | 0 | 1 | IncHI1B | Non-Mobilizable | 0 | 0 | 1 | 1 | 11 | - |
| 23 | pCR-HvKP4-VIR | CP040540.1 | 178154 | 1 | 1 | 1 | 1 | 1 | 1 | 1 | 0 | 0 | 0 | 0 | 0 | 0 | 1 | IncFIB and IncHI1B | Non-Mobilizable | 0 | 0 | 1 | 1 | 11 | - |
| 24 | pCR-HvKP1-VIR | CP040534.1 | 177790 | 1 | 1 | 1 | 1 | 1 | 1 | 1 | 0 | 0 | 0 | 0 | 0 | 0 | 1 | IncFIB and IncHI1B | Non-Mobilizable | 0 | 0 | 1 | 1 | 11 | - |
| 25 | pCR-HvKP5-VIR | CP040546.1 | 177694 | 1 | 1 | 1 | 1 | 1 | 1 | 1 | 0 | 0 | 0 | 0 | 0 | 0 | 1 | IncFIB and IncHI1B | Non-Mobilizable | 0 | 0 | 1 | 1 | 11 | - |
| 26 | p3-L491 | CP029228.2 | 177614 | 1 | 1 | 1 | 1 | 1 | 1 | 1 | 0 | 0 | 0 | 0 | 0 | 0 | 1 | IncFIB and IncHI1B | Non-Mobilizable | 0 | 0 | 1 | 1 | 11 | - |
| 27 | p2_L382 | CP033961.1 | 145683 | 1 | 1 | 1 | 1 | 1 | 1 | 1 | 0 | 0 | 0 | 0 | 0 | 0 | 1 | IncFIB and IncHI1B | Non-Mobilizable | 0 | 0 | 1 | 1 | 11 | - |
| 28 | p2-L491 | CP029227.1 | 130876 | 1 | 1 | 1 | 1 | 1 | 1 | 1 | 0 | 0 | 0 | 0 | 0 | 0 | 1 | IncHI1B | Non-Mobilizable | 0 | 0 | 0 | 1 | 11 | - |
| 29 | Unnamed2 | CP040994.1 | 96085 | 0 | 0 | 0 | 0 | 0 | 0 | 0 | 1 | 1 | 0 | 0 | 0 | 1 | 0 | IncFIA and IncFIB | Non-Mobilizable | 0 | 0 | 1 | 1 | 11 | - |
| 30 | unnamed1 | CP034046.1 | 345775 | 1 | 1 | 1 | 1 | 1 | 1 | 1 | 0 | 0 | 0 | 0 | 0 | 0 | 0 | IncFIB and IncHI1B | Conjugative | 1 | 1 | 1 | 1 | 15 | *bla*_TEM-1_*, bla*_CTX-M-15_*, aph(3')-Ia, sat-2* *, bla*_SHV-5_*, sul1, qacEdelta1, aadA, aac(3)-IIe, dfrA1* |
| 31 | unnamed1 | CP034054.1 | 299188 | 1 | 1 | 1 | 1 | 1 | 1 | 1 | 0 | 0 | 0 | 0 | 0 | 0 | 1 | IncFIB and IncHI1B | Non-Mobilizable | 0 | 0 | 1 | 1 | 15 | *bla*_TEM-1_*, aac(3)-IIe* |
| 32 | p17-16-vir | MK191024.1 | 290451 | 1 | 1 | 1 | 1 | 1 | 1 | 1 | 0 | 0 | 0 | 0 | 0 | 0 | 1 | IncFIB and IncHI1B | Conjugative | 1 | 1 | 1 | 1 | 15 | *qnrB4, bla_DHA-1_, sul1, armA, msrE, mphE, bla*_SHV-11_ |
| 33 | pKpvST15 | CP040595.1 | 277162 | 1 | 1 | 1 | 1 | 1 | 1 | 1 | 0 | 0 | 0 | 0 | 0 | 0 | 0 | IncFIB and IncHI1B | Conjugative | 1 | 1 | 1 | 1 | 15 | *bla*_CMY-6_*, qacEdelta1,* *rmtC, aac(6')-Ib10, sul1* |
| 34 | p2579_1 | MK649822.1 | 182805 | 1 | 1 | 1 | 1 | 1 | 1 | 1 | 0 | 0 | 0 | 0 | 0 | 0 | 1 | IncFIB and IncHI1B | Non-Mobilizable | 0 | 0 | 1 | 1 | 15 | - |
| 35 | pRJA166b | CP019049.1 | 228613 | 1 | 1 | 1 | 1 | 1 | 1 | 1 | 1 | 1 | 1 | 1 | 1 | 1 | 1 | IncFIB and IncHI1B | Non-Mobilizable | 0 | 0 | 1 | 1 | 23 | - |
| 36 | pAP855 | CP035384.1 | 357837 | 1 | 1 | 1 | 1 | 1 | 1 | 1 | 1 | 1 | 1 | 1 | 1 | 1 | 1 | IncFIB IncFII, and IncHI1B | Conjugative | 1 | 1 | 1 | 1 | 23 | - |
| 37 | pKP70-2 | MF398271.1 | 238153 | 1 | 1 | 1 | 1 | 1 | 1 | 1 | 1 | 1 | 1 | 1 | 1 | 1 | 1 | IncFIB IncFII, and IncHI1B | Non-Mobilizable | 0 | 0 | 1 | 1 | 23 | *dfrA14, bla*_KPC-2_ |
| 38 | pSGH10 | CP025081.1 | 231583 | 1 | 1 | 1 | 1 | 1 | 1 | 1 | 1 | 1 | 1 | 1 | 1 | 1 | 1 | IncFIB and IncHI1B | Non-Mobilizable | 0 | 0 | 1 | 1 | 23 | - |
| 39 | pR210-2-vir | CP034083.1 | 229648 | 1 | 1 | 1 | 1 | 1 | 1 | 1 | 1 | 1 | 1 | 1 | 1 | 1 | 1 | IncFIB and IncHI1B | Non-Mobilizable | 0 | 0 | 1 | 1 | 23 | - |
| 40 | pRJF999 | CP014011.1 | 228907 | 1 | 1 | 1 | 1 | 1 | 1 | 1 | 1 | 1 | 1 | 1 | 1 | 1 | 1 | IncFIB and IncHI1B | Non-Mobilizable | 0 | 0 | 1 | 1 | 23 | - |
| 41 | pTHC11-1 | AP019549.1 | 228290 | 1 | 1 | 1 | 1 | 1 | 1 | 1 | 1 | 1 | 1 | 1 | 1 | 1 | 1 | IncFIB and IncHI1B | Non-Mobilizable | 0 | 0 | 1 | 1 | 23 | - |
| 42 | pDHQP1701672_hv | CP037743.1 | 227239 | 1 | 1 | 1 | 1 | 1 | 1 | 1 | 1 | 1 | 1 | 1 | 1 | 1 | 1 | IncFIB and IncHI1B | Non-Mobilizable | 0 | 0 | 1 | 1 | 23 | - |
| 43 | pK2044 | CP026012.1 | 224152 | 1 | 1 | 1 | 1 | 1 | 1 | 1 | 1 | 1 | 1 | 1 | 1 | 1 | 1 | IncFIB and IncHI1B | Non-Mobilizable | 0 | 0 | 1 | 1 | 23 | - |
| 44 | Unnamed1 | CP035906.1 | 216620 | 1 | 1 | 1 | 1 | 1 | 1 | 1 | 1 | 1 | 1 | 1 | 1 | 1 | 1 | IncFIB | Non-Mobilizable | 0 | 0 | 1 | 1 | 23 | *catI* |
| 45 | pvirulence_VBA34918 | CP036191.1 | 216586 | 1 | 1 | 1 | 1 | 1 | 1 | 1 | 1 | 1 | 1 | 1 | 1 | 1 | 1 | IncFIB | Non-Mobilizable | 0 | 0 | 1 | 1 | 23 | *catI* |
| 46 | unnamed | CP016815.1 | 212770 | 1 | 1 | 1 | 1 | 1 | 1 | 1 | 1 | 1 | 1 | 1 | 1 | 1 | 1 | IncFIB and IncHI1B | Non-Mobilizable | 0 | 0 | 1 | 1 | 23 | - |
| 47 | p11492-CTXM | CP026022.1 | 193176 | 1 | 1 | 1 | 1 | 1 | 1 | 1 | 1 | 1 | 1 | 1 | 1 | 1 | 1 | IncFIB and IncHI1B | Non-Mobilizable | 0 | 0 | 1 | 1 | 23 | *bla*_CTX-M-24_ |
| 48 | pKP6-1 | CP025634.1 | 172768 | 1 | 1 | 1 | 1 | 1 | 1 | 1 | 1 | 1 | 1 | 1 | 1 | 1 | 1 | IncFIB and IncHI1B | Non-Mobilizable | 0 | 0 | 1 | 0 | 23 | - |
| 49 | pKP | CP025632.1 | 169967 | 1 | 1 | 1 | 1 | 1 | 1 | 1 | 0 | 1 | 1 | 1 | 1 | 1 | 1 | IncFIB and IncHI1B | Non-Mobilizable | 0 | 0 | 1 | 1 | 23 | - |
| 50 | pKp_Goe_414-2 | CP018338.1 | 202175 | 1 | 1 | 1 | 1 | 1 | 1 | 1 | 1 | 1 | 1 | 0 | 0 | 0 | 1 | IncFIB | Non-Mobilizable | 0 | 0 | 1 | 1 | 23 | - |
| 51 | pVir_020079 | CP029383.2 | 178741 | 1 | 1 | 1 | 1 | 1 | 1 | 1 | 1 | 1 | 1 | 0 | 0 | 0 | 0 | IncFIB and IncHI1B | Non-Mobilizable | 0 | 0 | 1 | 1 | 23 | - |
| 52 | pVir-SCNJ1 | MK715436.1 | 211807 | 1 | 1 | 1 | 1 | 1 | 1 | 1 | 1 | 1 | 1 | 1 | 1 | 1 | 1 | IncFIB | Non-Mobilizable | 0 | 0 | 1 | 1 | 29 | - |
| 53 | pTK421_2 | CP045692.1 | 127353 | 0 | 1 | 1 | 1 | 1 | 1 | 1 | 1 | 1 | 1 | 1 | 1 | 1 | 0 | IncFIB and IncR | Mobilizable | 1 | 1 | 1 | 1 | 34 | - |
| 54 | pVir_095132 | CP028390.2 | 208166 | 1 | 1 | 1 | 1 | 1 | 1 | 1 | 1 | 1 | 1 | 1 | 1 | 1 | 1 | IncFIB and IncHI1B | Non-Mobilizable | 0 | 0 | 1 | 1 | 36 | - |
| 55 | pMR0617aac | CP024459.1 | 211313 | 0 | 0 | 1 | 1 | 1 | 1 | 1 | 0 | 0 | 0 | 0 | 0 | 0 | 0 | IncFIB and IncFII | Conjugative | 1 | 1 | 1 | 1 | 45 | *sul2, aph(3'')-Ib, aph(6)-Id, tet(A), aac(3)-IIe* |
| 56 | pPUTH1 | CP024708.1 | 237216 | 1 | 1 | 1 | 1 | 1 | 1 | 1 | 1 | 1 | 1 | 1 | 1 | 1 | 1 | IncFIB and IncHI1B | Non-Mobilizable | 0 | 0 | 1 | 1 | 65 | - |
| 57 | pLVPK | AY378100.1 | 219385 | 1 | 1 | 1 | 1 | 1 | 1 | 1 | 1 | 1 | 1 | 1 | 1 | 1 | 1 | IncFIB and IncHI1B | Non-Mobilizable | 0 | 0 | 1 | 1 | 86 | - |
| 58 | phvKP060 | CP034776.1 | 218147 | 1 | 1 | 1 | 1 | 1 | 1 | 1 | 1 | 1 | 1 | 1 | 1 | 1 | 1 | IncFIB and IncHI1B | Non-Mobilizable | 0 | 0 | 1 | 1 | 86 | - |
| 59 | p1 | CP026587.1 | 215697 | 1 | 1 | 1 | 1 | 1 | 1 | 1 | 1 | 1 | 1 | 1 | 1 | 1 | 1 | IncFIB and IncHI1B | Non-Mobilizable | 0 | 0 | 1 | 1 | 86 | - |
| 60 | pINF237_01-VP | CP032834.1 | 133713 | 0 | 1 | 1 | 1 | 1 | 1 | 1 | 1 | 0 | 0 | 0 | 0 | 0 | 0 | IncFIB | Mobilizable | 0 | 1 | 1 | 1 | 90 | - |
| 61 | pKpvST101 | CP031369.1 | 292735 | 1 | 1 | 1 | 1 | 0 | 1 | 1 | 1 | 1 | 0 | 0 | 0 | 0 | 1 | IncFIB and IncFII | Conjugative | 1 | 1 | 1 | 1 | 101 | *sul1, aph(6)-Id* |
| 62 | pKpvST147B_virulence | CP040726.1 | 339117 | 1 | 1 | 1 | 1 | 1 | 1 | 1 | 1 | 1 | 1 | 0 | 0 | 0 | 1 | IncFIB and IncHI1B | Conjugative | 1 | 1 | 1 | 1 | 147 | *bla*_CTX-M-15_*, sul2, msrE, mphE, dfrA5, sul1, mphA, qacEdelta1* |
| 63 | pKP1677 | MN058044.1 | 202517 | 1 | 1 | 1 | 1 | 1 | 1 | 1 | 1 | 1 | 1 | 1 | 1 | 1 | 1 | IncFIB | Non-Mobilizable | 0 | 0 | 1 | 1 | 268 | - |
| 64 | pKP1692 | CP041024.1 | 202516 | 1 | 1 | 1 | 1 | 1 | 1 | 1 | 1 | 1 | 1 | 1 | 1 | 1 | 1 | IncFIB | Non-Mobilizable | 0 | 0 | 1 | 1 | 268 | - |
| 65 | pTMTA63631 | AP019666.1 | 166700 | 0 | 0 | 0 | 0 | 0 | 0 | 0 | 1 | 1 | 1 | 1 | 1 | 1 | 1 | IncFIB | Non-Mobilizable | 0 | 0 | 0 | 1 | 268 | - |
| 66 | p16114547_1 | MK649829.1 | 187989 | 0 | 0 | 1 | 1 | 1 | 1 | 1 | 0 | 0 | 0 | 0 | 0 | 0 | 0 | IncFIB and IncFII | Conjugative | 1 | 1 | 1 | 1 | 290 | *bla*_TEM-1_*, qnrS1, tet(A)* |
| 67 | pRJF293 | CP014009.1 | 224263 | 1 | 1 | 1 | 1 | 1 | 1 | 1 | 1 | 1 | 1 | 1 | 1 | 1 | 1 | IncFIB and IncHI1B | Non-Mobilizable | 0 | 0 | 1 | 1 | 374 | - |
| 68 | pKCTC2242 | CP002911.1 | 202852 | 1 | 1 | 1 | 1 | 1 | 1 | 1 | 1 | 1 | 1 | 1 | 1 | 1 | 0 | IncFIB and IncHI1B | Non-Mobilizable | 0 | 0 | 1 | 1 | 375 | - |
| 69 | pKC-Pl-HB1 | CP030924.1 | 196000 | 1 | 1 | 1 | 1 | 1 | 1 | 1 | 1 | 1 | 1 | 1 | 1 | 1 | 0 | IncFIB and IncHI1B | Non-Mobilizable | 0 | 0 | 1 | 1 | 375 | - |
| 70 | pKpvST383L | CP034201.1 | 374071 | 1 | 1 | 1 | 1 | 1 | 1 | 1 | 1 | 1 | 0 | 0 | 0 | 0 | 1 | IncFIB and IncHI1B | Conjugative | 0 | 0 | 1 | 1 | 383 | *bla*_NDM-1_ |
| 71 | pGN-2 | CP019161.1 | 261986 | 1 | 1 | 1 | 1 | 1 | 1 | 1 | 1 | 1 | 1 | 1 | 1 | 1 | 1 | IncFIB and IncHI1B | Conjugative | 1 | 1 | 1 | 1 | 485 | - |
| 72 | pKP8-2 | CP025638.1 | 153586 | 1 | 1 | 1 | 1 | 1 | 1 | 1 | 1 | 1 | 1 | 1 | 1 | 1 | 1 | IncFIB and IncFII | Conjugative | 1 | 1 | 1 | 1 | 485 | - |
| 73 | p1675479_1 | MK649828.1 | 167922 | 0 | 0 | 1 | 1 | 1 | 1 | 1 | 0 | 0 | 0 | 0 | 0 | 0 | 0 | IncFIB and IncFII | Conjugative | 1 | 1 | 1 | 1 | 945 | *sul2, aph (6)-Id, aph(3'')-Ib* |
| 74 | p11420-HVKP | CP026024.1 | 229796 | 1 | 1 | 1 | 1 | 1 | 1 | 1 | 1 | 1 | 1 | 1 | 1 | 1 | 1 | IncFIB and IncHI1B | Non-Mobilizable | 0 | 0 | 1 | 1 | 1265 | - |
| 75 | pSC7-vir | CP030270.1 | 236809 | 1 | 1 | 1 | 1 | 1 | 1 | 1 | 1 | 1 | 1 | 1 | 1 | 1 | 1 | IncFIB | Non-Mobilizable | 0 | 0 | 1 | 1 | 1660 | *sul1, qacEdelta1, aadA, cmlA1, ant (3'')-IIa* |
| 76 | pKP7 | CP025642.1 | 197806 | 1 | 1 | 1 | 1 | 1 | 1 | 1 | 1 | 1 | 1 | 1 | 1 | 1 | 1 | IncFIB and IncHI1B | Non-Mobilizable | 0 | 0 | 1 | 1 | 1941 | - |
| 77 | pBA813_1 | MK649825.1 | 273676 | 1 | 1 | 1 | 1 | 1 | 1 | 1 | 0 | 0 | 0 | 0 | 0 | 0 | 0 | IncFIB and IncHI1B | Conjugative | 1 | 1 | 1 | 1 | 2096 | *bla*_CTX-M-15_*, bla*_TEM-1,_ *tet(D), dfrA14, bla*_OXA-1_*, mphE, msrE, armA, sul1, qacEdelta1, aadA2, dfrA12, aac(6')-Ib-cr6* |
| 78 | pF81_2 | CP026166.1 | 211437 | 1 | 1 | 1 | 1 | 1 | 1 | 1 | 1 | 1 | 1 | 1 | 1 | 1 | 1 | IncFIB | Non-Mobilizable | 0 | 0 | 1 | 1 | 3685 | - |
| 79 | pKpvK54 | CP023135.2 | 211454 | 1 | 1 | 1 | 1 | 1 | 1 | 1 | 1 | 1 | 1 | 1 | 1 | 1 | 1 | IncFIB | Non-Mobilizable | 0 | 0 | 1 | 1 | 3813 | - |

***UD:** undetermined; **Conjugative:** positive for all four conjugal constituents including oriT, relaxase, type IV coupling protein (T4CP) and type IV secretion system (T4SS); **Mobilizable:** only lacked oriT of four conjugal constituents
