# Supplementary material for: Comparative in silico characterization of Klebsiella pneumoniae hypervirulent plasmids and their antimicrobial resistance genes
Source: Ann Clin Microbiol Antimicrob. 2022 Jun 2;21:23. doi: 10.1186/s12941-022-00514-6 (PMC9161459; doi:10.1186/s12941-022-00514-6)
Supplement: Supplementary file 2 — Additional file 2: Table S2. The information of prophages found in hypervirulent plasmids of Klebsiella pneumoniae. [file 12941_2022_514_MOESM2_ESM.docx]

**Table S2**. The information of prophages found in hypervirulent plasmids of K. pneumoniae.

| Plasmids | Detected prophages^*^ | Detected ARGs | ARGs Related ISs |
| --- | --- | --- | --- |
| MK181633.1 | Bacill_Shanette: incomplete | *bla*_CTX-M-15_, *bla*_TEM-1_, *bla*_SHV-11_, *mph* and *aph(3')-Ia* | IS*1380*, IS*6* and IS*110* |
|  | Escher_Av: incomplete | *-* |  |
|  | Escher_RCS47: incomplete | *-* |  |
|  | Salmon_SJ46: incomplete | *-* |  |
| MK649825.1 | Escher_RCS47: questionable  Pseudo_nickie: incomplete  Salmon_SJ46: incomplete | *dfrA12* | - |
| CP034046.1 | Entero_VT2_Sakai: incomplete | - | IS*5* and IS*1380* |
|  | Salmon_SJ46: incomplete | *bla*_CTX-M-15_, *bla*_TEM-1_ and *aac(3)-IIe* |  |
| MK191024.1 | Bacill_Shanette: incomplete  Escher_Av: incomplete | ND | - |
| CP034054.1 | Cronob_vB_CsaP: incomplete | - | IS*14*, IS*13* and DDE  transposase |
|  | Escher_Av: incomplete | *bla*_TEM-1_ and *aac(3)-IIe* |  |
|  | Salmon_SJ46: incomplete | *-* |  |
| CP025462.1 | Burkho_KL3: incomplete  Entero_If1: incomplete  Escher_RCS47: incomplete  Entero_mEp390: incomplete | ND | - |
| MF398271.1 | Escher_RCS47: intact  Escher_SH2026St: questionable  ntero_mEpX1: incomplete | ND | - |
| CP026022.1 | Escher_SH2026St: questionable  Proteu_Privateer: incomplete | ND | - |
| CP040726.1 | Entero_mEp237: incomplete | *bla*_CTX-M-15_ | IS*1380* |
|  | Gordon_Smoothie: incomplete  Escher_Av: incomplete | *-* |  |
| MK649829.1 | Microb_Hendrix: questionable | - | IS*6* |
|  | Escher_RCS47: incomplete | *tet(A) bla*_TEM-1_ and *qnrS1* |  |
|  | Salmon_SJ46: incomplete | *-* |  |
| CP040595.1 | Escher_SH2026S: incomplete | *-* | IS*6* and IS*110* |
|  | Salmon_SJ46: incomplete | *aac(6')-Ib10* |  |
| CP028791.1 | Acinet_vB_AbaM_ME3: intact | - |  |
|  | Escher_RCS47: incomplete | *catB3*, *aa(6')-Ib-cr6* and *bla*_OXA-1_, | IS*6* and *IS110* |
|  | Entero_mEp237: incomplete | *-* |  |
|  | Salmon_SJ46: incomplete | *-* |  |
|  | Escher_RCS47: questionable  Burkho_KL3: incomplete  Bacill_Shanette | ND |  |
| MK413722.1 |  |  | - |

* Intact, incomplete, or questionable prophage: The predicted region contains an intact prophage based on the criteria. If the region's total score is less than 70, it is marked incomplete; if it is between 70 to 90, it is marked questionable; if it is greater than 90, it is marked intact.

*ND: Not detected.
