# Supplementary figures and images for: Comparative in silico characterization of Klebsiella pneumoniae hypervirulent plasmids and their antimicrobial resistance genes
Source: Ann Clin Microbiol Antimicrob. 2022 Jun 2;21:23. doi: 10.1186/s12941-022-00514-6 (PMC9161459; doi:10.1186/s12941-022-00514-6)

## Slide 1
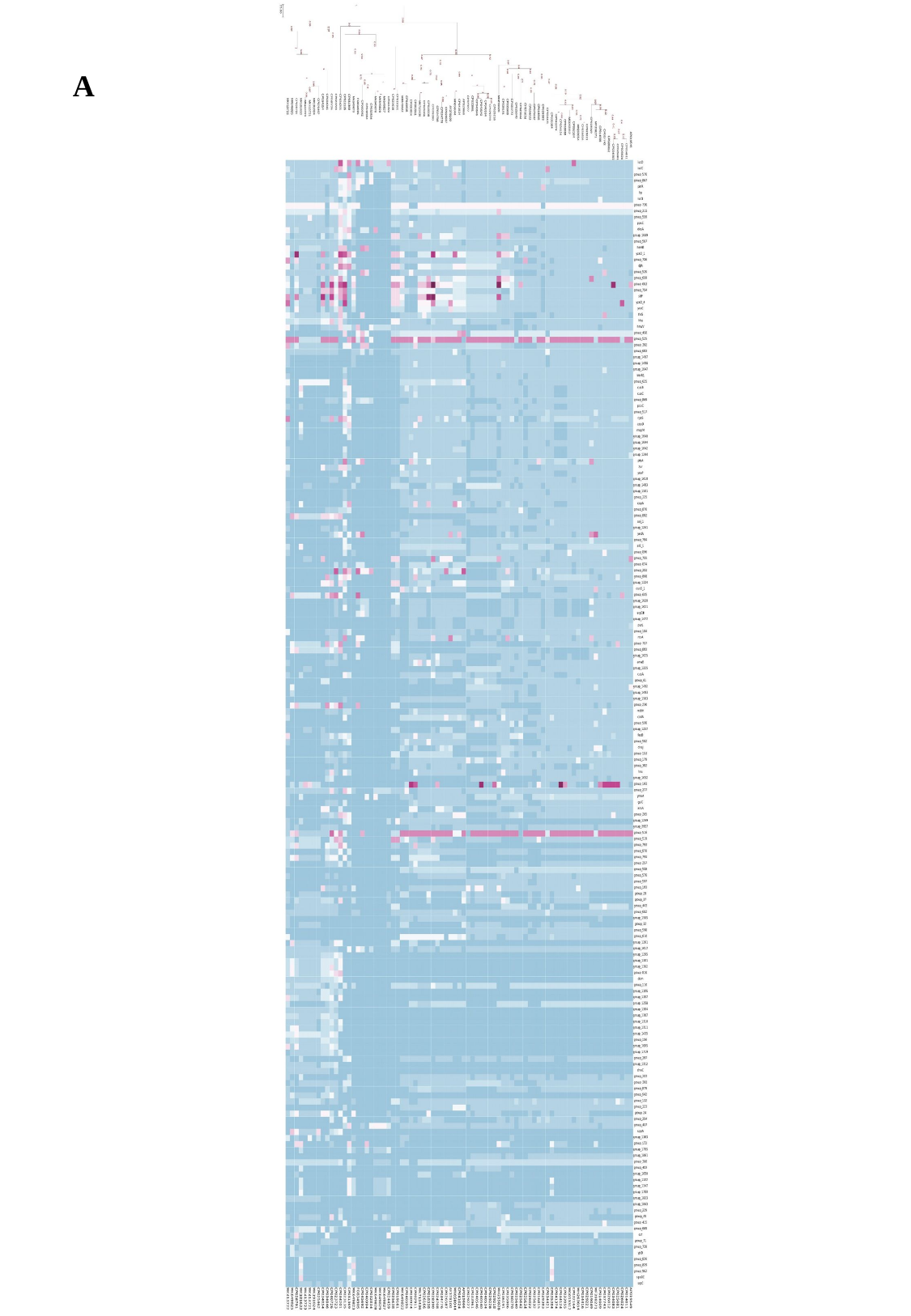

A

## Slide 2
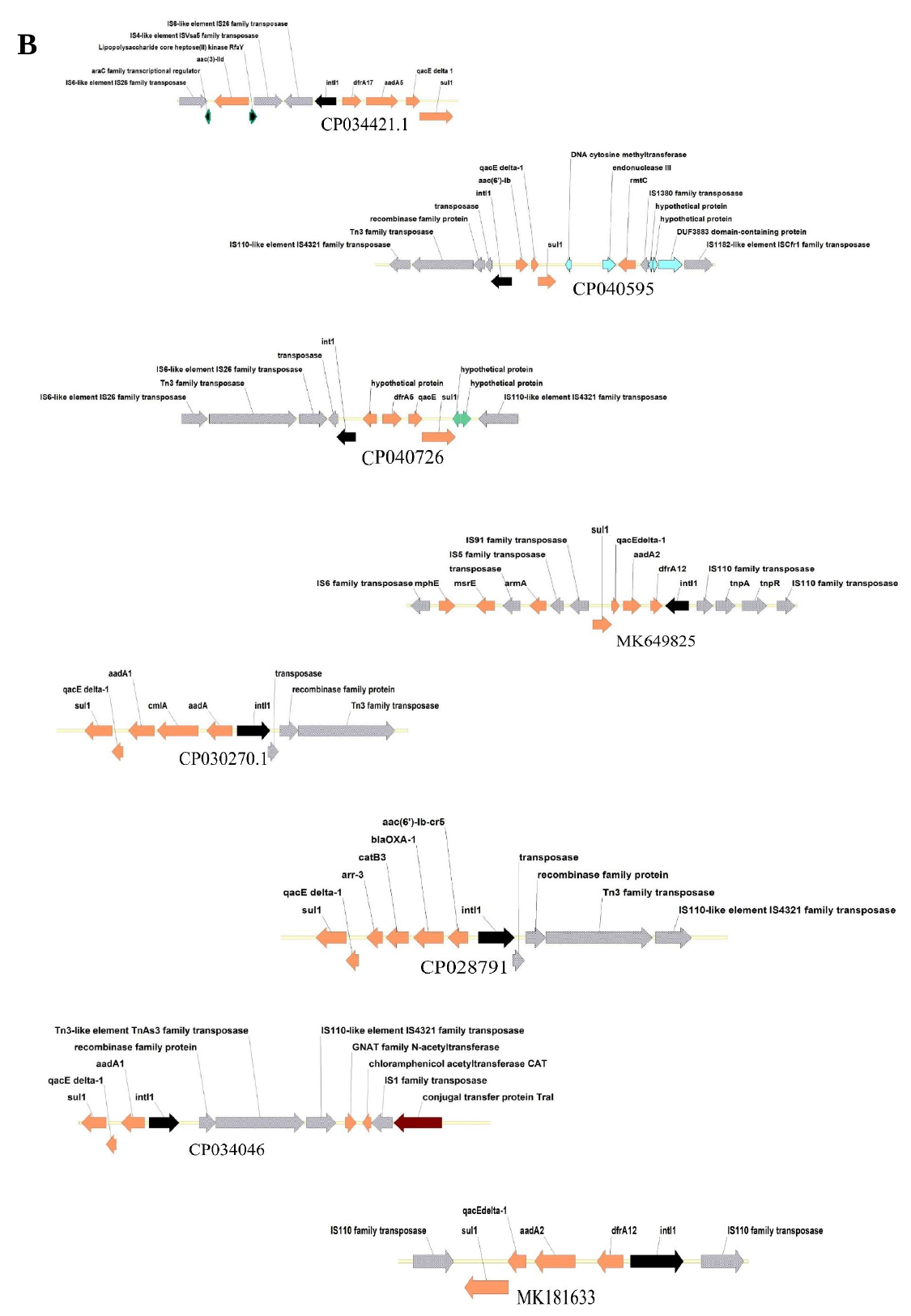

B

Supplement: Supplementary file 3 — Additional file 3. Fig. 1. The ARGs cassettes surrounding class 1 integron in Klebsiella pneumoniae hypervirulent plasmids. Fig. 2. The phylogenic tree and heatmap of 79 hypervirulent Klebsiella pneumoniae plasmids based on 185 loci. [file 12941_2022_514_MOESM3_ESM.pptx]
